# School Cramming

**Published:** 1892-11

**Authors:** 


### SCHOOL CRAMMING.

A writer who signs herself Jennie Wren, in the Oakland *Morning Times*, says :—

I have one bright little friend who came home from school every night with brilliant eyes, flushed cheeks, and every muscle twitching, carrying under her arm a great pile of books for home study. She was sensitive and peevish, her voice shrill and excited, her eyelids and lips never still. At my urgent request her mother took her out of school for six months, furnishing her enough quiet duties to preserve discipline, and keeping her out-of-doors a great deal. The change is simply marvelous. Still a very nervous child, her eyelids stopped twitching; her voice is softer, her temper sweeter, and the work she has to do is well done, simply because she is not kept under continual restraint.

I know another girl, a quiet, slow, earnest little soul, who nearly studied herself to death last term. Long before the last of May she was pale and languid, her movements were heavy, and every fresh exertion was accompanied by an unconscious, pathetic little sigh. Her mother became very much worried about her, and contemplated giving her a year's rest, which nearly broke the little maiden's heart, because she would drop behind her class.

Commenting on this, a writer in the *Pacific Health Journal* makes the following sensible remarks :—

The above is not overdrawn. The fact of the case is that the high pressure of nineteenth century life is developing a very nervous class of children, especially in our cities, children with large brains and weak, puny bodies, incapable of bearing the strain which their excessive mentalitv, aided by the school cramming process, puts upon them. It was once so rare an occurrence in the world for a son to die before his father that inspired history of marvelous brevity considers it worthy of note. (Gen. 11 : 28.) But those who die now before manhood and womanhood are legion ; and many of those who do not die, live miserable lives, with broken down constitutions, weak, nervous, hopeless.

Of course all this is not due to the schools alone. Deficient sleep and irregular hours at home are heavy weights upon the little boy and girl. The more highly organized and sensitive the nervous system of the child, the more sleep is needed, but it is just this class that gets the least. They do not get sleepy until late at night, their overwrought nerves do not allow it, and the careless or unwise parent yields to the entreaties of the "wide-awake," and it (she, generally) is allowed to remain up till nine, ten, or even eleven o'clock, to be aroused in the morning peevish and fretful because of insufficient sleep. A nervous girl ought to have on an average eight or nine hours' sleep till fifteen years of age, and many times an hour or two extra is time well invested. We do not mean that they should be in bed simply ; they should put that time in in sleep. It is not a good thing for children to remain in bed except to sleep.

Another matter which increases the odds against the child is deficient food. We do not imply that parents generally do not furnish their children food enough in quantity, for they do, but it is frequently a mighty poor quality. The child arises in the morning after a deficient night's rest, weak, nervous, irritable, with dark rings around the eyes, and with little or no appetite. Nothing tastes good unless it is fried potatoes, dyspeptic toast (toast which causes dyspepsia, we mean) warm tea or coffee, white bread and butter, with pickles, perhaps for a

"relish." We have seen just such breakfasts as that set before children. It is simply abominable. Give the child a light supper, send her to bed early, place before her some nice graham gems, wheaten rolls, well cooked mush and good milk, food which will build up and strengthen every part of the system. The child may not like plain food at first, but it will pay to educate her so that she will possess such an appetite as to like any thing which is good and wholesome.

But we did not design to say all of this when we began our comments on "Jenny Wren's" appeal. We do not believe in this "cramming process" of our schools as applied to the weak, sickly children of this generation, nor do we believe it is just the best thing for the strong children. However, the schools are not wholly at fault. Let the parents do all they can to fit their children for the heavy task of life by seeing that the child has a healthy, strong physical basis upon which, or in which, to build his mental training. With this should come, of course, a sound moral training. Plenty of sleep, plenty of open air exercise, good ventilation, and good food at regular intervals, are among the physical *desiderata* for such an object.

